# Dissection of the genetic basis of oil content in Chinese peanut cultivars through association mapping

**DOI:** 10.1186/s12863-020-00863-1

**Published:** 2020-06-08

**Authors:** Nian Liu, Li Huang, Weigang Chen, Bei Wu, Manish K. Pandey, Huaiyong Luo, Xiaojing Zhou, Jianbin Guo, Haiwen Chen, Dongxin Huai, Yuning Chen, Yong Lei, Boshou Liao, Xiaoping Ren, Rajeev K. Varshney, Huifang Jiang

**Affiliations:** 1 Key Laboratory of Biology and Genetic Improvement of Oil Crops, Ministry of Agriculture and Rural Affairs, Oil Crops Research Institute of the Chinese Academy of Agricultural Sciences, Wuhan, 430062 People’s Republic of China; 2 International Crops Research Institute for the Semi-Arid Tropics (ICRISAT), 502324, Hyderabad, India

**Keywords:** Peanut, Oil content, Association analysis, Geographic region

## Abstract

**Background:**

Peanut is one of the primary sources for vegetable oil worldwide, and enhancing oil content is the main objective in several peanut breeding programs of the world. Tightly linked markers are required for faster development of high oil content peanut varieties through genomics-assisted breeding (GAB), and association mapping is one of the promising approaches for discovery of such associated markers.

**Results:**

An association mapping panel consisting of 292 peanut varieties extensively distributed in China was phenotyped for oil content and genotyped with 583 polymorphic SSR markers. These markers amplified 3663 alleles with an average of 6.28 alleles per locus. The structure, phylogenetic relationship, and principal component analysis (PCA) indicated two subgroups majorly differentiating based on geographic regions. Genome-wide association analysis identified 12 associated markers including one (AGGS1014_2) highly stable association controlling up to 9.94% phenotypic variance explained (PVE) across multiple environments. Interestingly, the frequency of the favorable alleles for 12 associated markers showed a geographic difference. Two associated markers (AGGS1014_2 and AHGS0798) with 6.90–9.94% PVE were verified to enhance oil content in an independent RIL population and also indicated selection during the breeding program.

**Conclusion:**

This study provided insights into the genetic basis of oil content in peanut and verified highly associated two SSR markers to facilitate marker-assisted selection for developing high-oil content breeding peanut varieties.

## Background

Cultivated peanut or groundnut (*Arachis hypogaea* L.) is one of the most important oilseed crops worldwide which sooner may gain the status of food crop in the near future because of diverse consumption modes [1]. The global annual planting area is stands at 28.52 Mha which yielded, with the annual production of 45.95 Mt of peanut production in 2018 [2]. Despite China being the largest peanut producer in the world, the current production hardly meets the increasing domestic demands for peanut oil. High seed oil content is one of the most significant traits, and increase of just 1% oil content in popularly grown varieties results in 7% increase in economic benefit to the farmers and oil processing units. Thus, enhancing oil content is an important long-term objective in all the peanut breeding programs not only in China but in many other countries such as India.

Oil content is a polygenetic trait and is significantly influenced by the environment [3, 4]. In the current breeding programs, high oil content lines are selected based on the phenotyping data generated from multiple environments, which is low efficient and time-consuming process. Deployment of well-validated markers could accelerate the process and precision in genetic improvement [5–9]. Dissection of the genetic architecture underlying oil content is a prerequisite to deploying markers in high-oil content breeding programs. Triacylglycerol is the major form of storage oil in most plant seeds including peanut. Its biosynthetic pathway and the relevant genes have been extensively understood in the model plant *Arabidopsis* [10, 11]. But, the genetic mechanism of natural variation for oil biosynthesis remains poorly understood in peanut.

The available significant variation of oil content among peanut germplasm provides an opportunity to identify genomic regions controlling higher oil accumulation [12]. Using the bi-parental populations with various phenotypes, linkage analyses were performed to identify quantitative loci and linked markers in peanut. For instance, six and nine QTLs for oil content have been respectively identified in two different recombinant inbred line (RIL) populations [13]. Subsequently, three and eight QTLs were detected in an advanced backcross population and RIL population, respectively [14, 15]. Most recently, seven QTLs for oil content were detected in a RIL population, including one major and stable QTL with 10.14–27.19% phenotypic variance explained (PVE) [16]. Despite discovery of several QTLs for oil content, linkage analysis of oil content hardly fully reveals genetic variations for oil content in peanut due to the limited number of parental lines used in above mentioned studies. Compared with linkage analysis, association analysis, utilizing historical recombination in natural populations, facilities high-resolution mapping and testing of multiple-alleles in far less time [17, 18]. This genetic method has been successfully used to reveal the genetic basis of complex agronomic traits in multiple crops [19–22], however, this approach has so far been deployed in couple of studies in peanut [23, 24].

Cultivars and elite germplasm lines possessing desirable traits get preference by plant breeders in using them as trait source in almost all the genetic improvement programs as compared to wild relatives. Therefore, discovery of associated genomic regions and potential candidate genes in elite association panel have potential of faster application in ongoing breeding programs. Therefore, the present study used the Chinese peanut panel which consists of 222 cultivars, 55 breeding lines, and 15 landraces to study (1) genetic architecture, (2) elucidation of genetic basis of natural variation in peanut cultivars for oil content, and (3) development and validation of associated markers which could be used to enhance oil content through genomics-assisted breeding (GAB).

## Results

### Genetic diversity, population structure and linkage disequilibrium analysis

A total of 583 simple sequence repeats (SSR) polymorphic markers randomly distributed on the genome, were used to genotype the association mapping panel of 292 peanut accessions. The polymorphic markers produced 3663 alleles with an average of 6.28 alleles per locus ranging from 2 to 20 (Table 1 and Additional file 1: Table S1). The major allele frequency ranged from 0.15 to 0.98, with a mean value of 0.60. The average genetic diversity was 0.51 and ranged from 0.03 to 0.90. The polymorphic information content (PIC) ranged from 0.03 to 0.90, with an average of 0.45 (Additional file 1: Table S1). Of the 3663 alleles, 629 were unique alleles (allele frequency < 0.05%), 1471 allele were rare alleles (0.05% ≤ allele frequency < 5%), 1547 allele were polymorphic alleles (5% ≤ allele frequency < 95%), and 15 were fixed alleles (allele frequency ≥ 95%), with corresponding proportions of 17.17, 40.16, 42.23 and 0.41%, respectively (Additional file 1: Table S2).

**Table 1:** Statistic summary for population diversity

Population	Sample Size	Allele Number	Major Allele Frequence	Genetic Diversity	PIC
G1	161	5.36	0.72	0.40	0.36
G2	131	5.29	0.66	0.47	0.42
Total	292	6.28	0.61	0.51	0.45

**Fig. 1:**
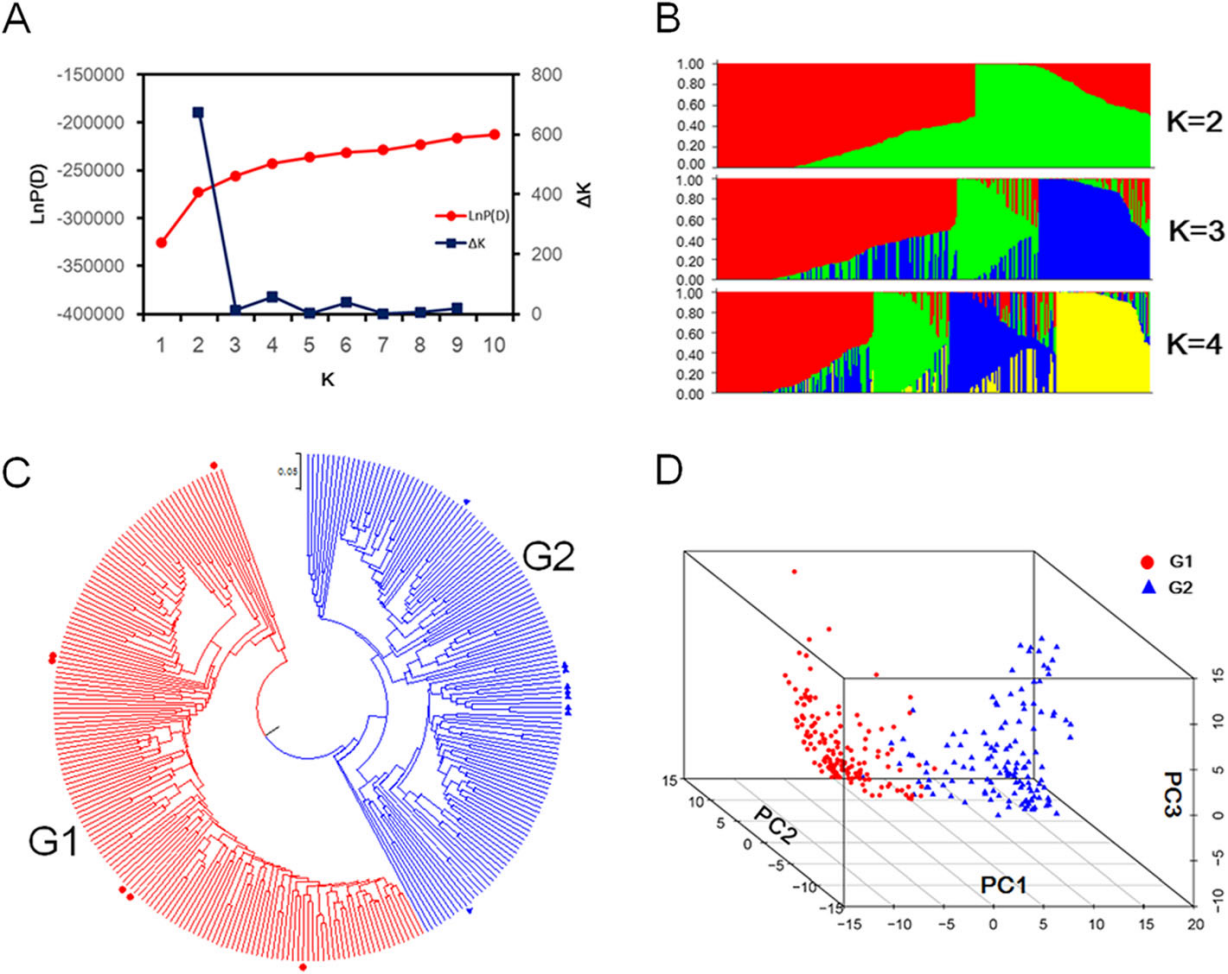
Population structure analysis in 292 peanut accessions. **a** Determination of optimal *K* based on LnP(D) and Δ*K* values. **b** the population structure in the peanut panel at *K* = 2, 3, 4. **c** Phylogenetic tree of the peanut panel based on Nei’s (1972) genetic distance. The 292 peanut accessions were grouped into two clusters G1 (red lines) and G2 (blue lines). The red dots represented the landraces belonging to Subsp. *hypogaea*, the blue triangles denoted the landraces belonging to Subsp. *fastigiata*. **d** Three-dimensional scatter plots of the first three principal components. The red dots represented cluster G1 in the phylogenetic tree. The blue triangles represented cluster G2 in the phylogenetic tree

The population structure analysis was performed using multi-allelic SSR genotyping data. The most significant change of the LnP(D) value was observed when parameter K increased from 1 to 2, and the highest ΔK value was obtained when K = 2 (Fig. 1a and b). The previously available information suggested two subgroups in the peanut panel and the results of this study on phylogenetic relationship and PCA analysis further proved that the 292 peanut accessions could be clearly divided into two subgroups (G1 and G2), which were consistent with the population structure results (Fig. 1c and d). All the landraces in G1 subgroup were subsp. *hypogaea*, while the landraces in G2 subgroup belonged to subsp. *fastigiata* (Fig. 1c). The pairwise *F*_*ST*_ value between the two subgroups was 0.16, and Nei’s (1972) genetic distance was 0.27. Compared with G1 subgroup, G2 had a relatively higher genetic diversity (0.47) and PIC value (0.36). However, the allele number per locus was higher in G1 than G2 (Table 1).

**Fig. 2:**
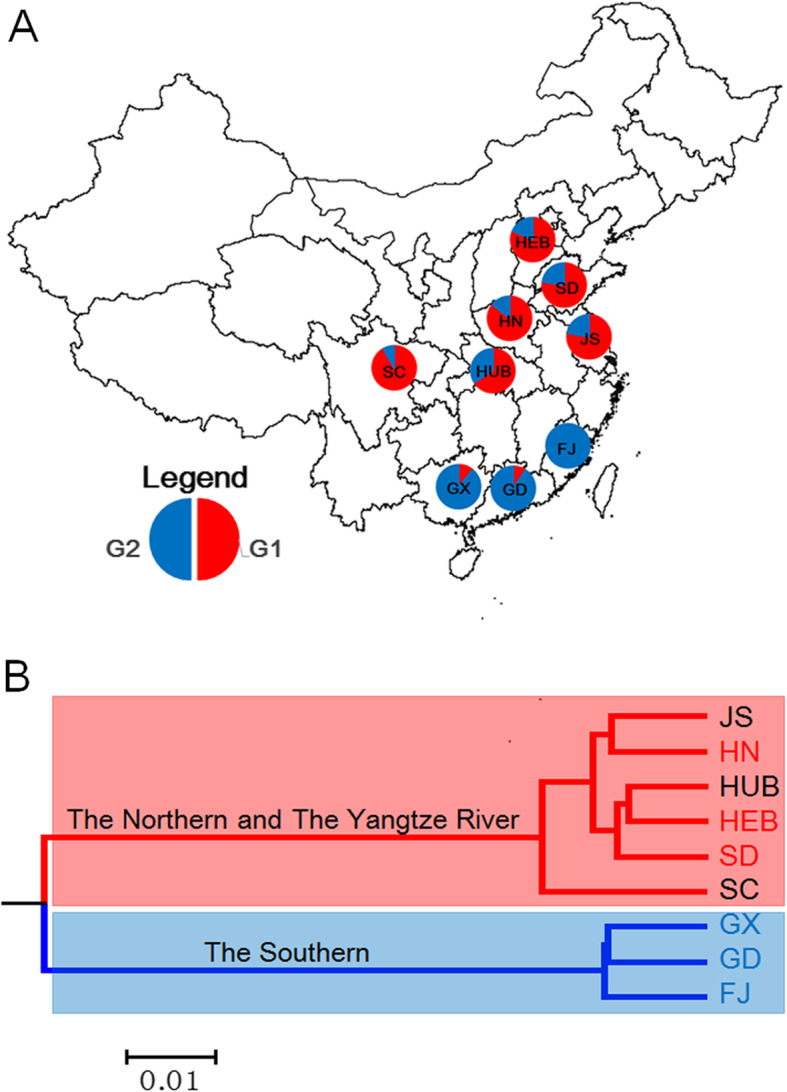
Geographical structure in the peanut panel. **a** The proportion of two groups G1 and G2 (Fig. 1c) in China. **b** Phylogenetic tree of the peanut accessions grouped by original provinces. HEB, Hebei province; SD, Shandong province; HN, Henan province; JS, Jiangsu province; HUB, Hubei province; SC, Sichuan province; FJ, Fujian province; GD, Guangdong province; GX, Guangxi province. HEB, SD, and HN belong to the Northern China. JS, HUB, and SC belong to the Yangtze River region in China. FJ, GD, and GX belong to the Southern China

The peanut association mapping panel consisted of cultivars from 17 provinces of China. Most accessions (93.2%) were distributed in nine provinces (Hebei, Shandong, Henan, Sichuan, Hubei, Jiangsu, Fujian, Guangdong, and Guangxi). The proportion of two subgroups in these provinces exhibited obvious differences (Fig. 2a). In Northern China (Hebei, Shandong, and Henan provinces), the proportion of G1 subgroup ranged from 77.42 to 85.71%. Similarly, the proportion of G1 ranged from 66.10 to 91.67% in peanut accessions distributed in the Yangtze River region (Sichuan, Hubei, and Jiangsu provinces). Whereas, the proportion of G1 subgroup were below 11.11% in Southern China (Fujian, Guangdong, and Guangxi provinces). It is suggested that genetic diversity was highly linked to geographic distribution. The phylogenetic tree showed that the peanut-distributed provinces could be clearly clustered into two clades (Fig. 2b). The provinces in Southern China (Fujian, Guangdong, and Guangxi) were clustered together, and the provinces from Northern China and the Yangtze River region were grouped into another clade.

The linkage disequilibrium (LD) was estimated using coefficients (*r*^*2*^) of 280 SSR markers mapped on 20 linkage groups [25]. The average *r*^*2*^ was 0.11 and almost 53.4% of the coefficients (*r*^*2*^) showed statistically significant (*P* < 0.01). The 95th percentile of distribution of all *r*^*2*^ between the unlinked marker-pairs, i.e., *r*^*2*^ = 0.28, was set as the background level. Since the average distance of pair combinations was below 1 cM with the *r*^*2*^ plot dropping to background level, the estimated LD decay in the peanut panel is 1 cM (Additional file 2: Fig. S1).

### Phenotypic variation for oil content among peanut accessions

The analysis was done for the phenotyping data generated on oil content for 292 Chinese peanut accessions from seeds harvested from four environments. The oil content among association panel ranged from 45.85 to 59.72% in 2015WH, 43.82 to 55.88% in 2016WH, 44.22 to 54.97% in 2017NC, and 45.11 to 56.69% in 2017WH, respectively (Table 2). The median values of oil content in four environments varied from 48.47 to 51.89%, and the standard deviation of phenotypic data ranged from 1.78 to 2.39 across four environments. Two elite cultivars (Zhonghua 15 and Yuhua 9326) which have superior yield potential, exhibited stably high-oil feature across four-environmental trials (average oil content > 55%). The continuous distributions of phenotypic values for peanut accessions were shown in Additional file 2: Fig. S2. The phenotypic data of the peanut panel in 2015WH, 2016WH, and 2017WH followed a normal distribution based on the Shapiro-Wilk normality test (Table 2). Variance analysis across four environmental trials showed that genotype, environment, and genotype × environment significantly influenced oil content at the *P* < 0.001 level (Additional file 2: Fig. S2). The broad sense heritability for oil content was evaluated to be 0.76 in the peanut panel.

**Table 2:** Phenotypic variation for oil content (%) for 292 peanut accessions across four environments

Env	Min (%)	Max (%)	Median (%)	SD	Kurt	Skew	w (Sig)	H^2^
2015WH	45.85	59.72	51.89	2.39	−0.04	0.22	1.00 (0.51)	0.76
2016WH	43.82	55.88	50.13	1.78	0.54	0.00	0.99 (0.36)	
2017NC	44.22	54.97	48.47	1.94	0.66	0.06	0.99 (0.04)	
2017WH	45.11	56.69	51.53	1.86	0.32	0.06	1.00 (0.50)	

*Env* environment, *SD* standard deviation, *Kurt* Kurtosis, *Skew* skewness, *w* Shariro-Wilk statistic value, *Sig* significance

We further studied phenotypic differences in the genetically highly diverse association mapping panel containing genotypes different geographic distributions in China. The oil content in the accessions from Northern China was statistically higher than that from Southern China in all the field trials. Similarly, the accessions from the Yangtze River region had higher oil content than the accessions from Southern China in 2016WH, 2017NC, and 2017WH. The phenotypic difference between Northern China and the Yangtze River region was not statistically significant in three of the four environments. Meanwhile, we also made a comparison among cultivated peanuts released at different times (Additional file 2: Fig. S3). In general, there was no obvious difference in oil content between cultivars released at different times.

### Association analysis for oil content

The Mixed linear model with K + Q matrix was used to perform association mapping with SSR-markers and the phenotypic data on oil content generated on 292 peanut accessions in four environments. The marker-trait association analysis identified two associated loci for 2015 WH environment, eight associated loci for 2016WH environment, three associated loci for 2017NC environment, and five associated loci for 2017WH environment. Twelve significantly associated loci at *P* < 0.00186 explained 4.54–9.94% phenotypic variance across four environments (Table 3 and Additional file 2: Fig. S4). Among them, AGGS1014_2 with up to 9.94% PVE had been repeatedly detected in multiple environments (2016WH, 2017NC, and 2017WH). These markers were widely distributed on nine linkage groups based on previously reported genetic maps (Additional file 1: Table S2). Physical position of associated markers were on 12.7 Mb of B01 (AGGS1014_2), 57.1 Mb of B07 (AGGS1081), 47.4 Mb of A03 (AGGS1149), 124.9 Mb of B06 (AHGS0798), 30.1 Mb of B08 (AHGS1388), 20.8 Mb of B06 (AHGS1431), 36.9 Mb of A04 (AHGS1679), 57.1 Mb of B07 (AHGS2053), 67.8 Mb of B07 (AHS0127), 119.6 Mb of A09 (pPGPseq8D9), 5.1 Mb of A10 (TC11B4_2), 35.5 Mb of A08 (TC9F10_2), respectively.

**Table 3:** Marker–trait associations across four environments for oil content

Marker	Environment	*F*-value	*P*-value	PVE(%)	Favorable allele
pPGPseq8D9	2017NC	13.22	3.29E-04	4.61	pPGPseq8D9-131 bp
TC9F10_2	2017WH	6.48	3.09E-04	7.59	TC9F10_2-256 bp
TC11B4_2	2017WH	4.08	1.43E-03	8.84	TC11B4_2-298 bp
AHGS1679	2017WH	5.95	6.04E-04	6.39	AHGS1679-293 bp
AGGS1149	2016WH	6.54	1.68E-03	4.54	AGGS1149-192 bp
AGGS1081	2016WH	5.47	1.15E-03	5.76	AGGS1081-201 bp
AGGS1014_2	2016WH	23.23	2.50E-06	9.94	AGGS1014_2-215 bp
	2017NC	14.89	1.45E-04	6.90	
	2017WH	21.43	5.90E-06	8.75	
AHGS2053	2016WH	6.29	3.81E-04	6.65	AHGS2053-256 bp
AHS0127	2016WH	10.04	6.13E-05	6.99	AHS0127-188 bp
AHGS1431	2016WH	9.11	1.52E-04	7.35	AHGS1431-260 bp
AHGS0798	2015WH	9.54	1.03E-04	7.28	AHGS0798-174 bp
AHGS1388	2016WH	8.84	1.94E-04	6.78	AHGS1388-304 bp

*PVE* phenotypic variance explained

**Fig. 3:**
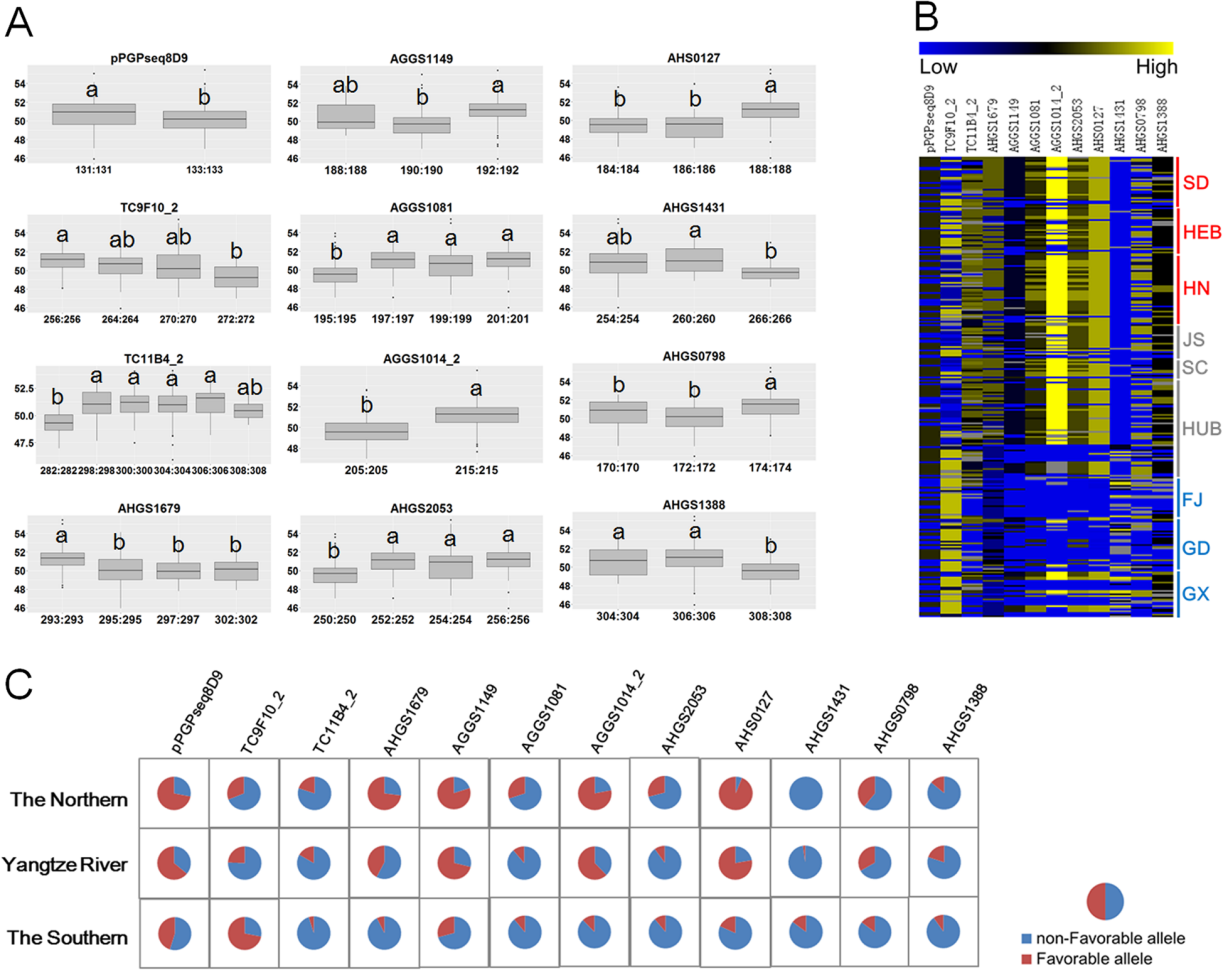
Phenotypic effect and geographic distribution of favorable alleles of trait-associated markers. **a** Comparison of accessions with different alleles based on average values of four environmental data. The boxes with different letters were significantly different according to Tukey’s Multiple Comparison Test (*P* < 0.05). **b** Overview of allelic effect of associated markers in accessions from nine provinces. The columns of heatmap denoted the association markers. The rows of heatmap represented accessions distributed on nine provinces. Each cell in the heatmap represented phenotypic effect of allele. **c** The spectrum of the most favorable allele frequencies in different geographic regions. HEB, Hebei province; SD, Shandong province; HN, Henan province; JS, Jiangsu province; HUB, Hubei province; SC, Sichuan province; FJ, Fujian province; GD, Guangdong province; GX, Guangxi province. HEB, SD, and HN belong to the Northern China. JS, HUB, and SC belong to the Yangtze River region in China. FJ, GD, and GX belong to the Southern China

The allelic number of these associated loci ranged from two (pPGPseq8D9 and AGGS1014_2) to six (TC11B4_2). The most favorable alleles which have the largest effect values included pPGPseq8D9-131 bp, TC9F10_2-256 bp, TC11B4_2-298 bp, AHGS1679-293 bp, AGGS1149-192 bp, AGGS1081-201 bp, AGGS1014_2-215 bp, AHGS2053-256 bp, AHS0127-188 bp, AHGS1431-260 bp, AHGS0798-174 bp, and AHGS1388-304 bp (Table 3, Additional file 1: Table S3). The accessions with different alleles showed statistically significant difference in a four-environment average of oil content (Fig. 3a). Compared with accessions in Southern China (FJ, GD, and GX), the genotypes from Northern China and Yangtze River (SD, HEB, HN, JS, SC, and HUB) carried more alleles with relatively high effect (Fig. 3b). Similarly, the frequencies of the most favorable alleles also showed geographic differences. For ten associated loci (pPGPseq8D9, TC11B4_2, AHGS1679, AGGS1149, AGGS1014_2, AHGS2053, AHGS0127, AHGS1431, AGHS0798, and AHGS1388), the most favorable allele frequency was highest in Northern China, the second-highest in the Yangtze River region, and lowest in Southern China (Fig. 3c). However, the most favorable allele frequencies were highest in Southern China for another two associated loci (TC9F10_2 and AHGS1431).

### Evaluation of RIL population and confirmation of associated markers

**Fig. 4:**
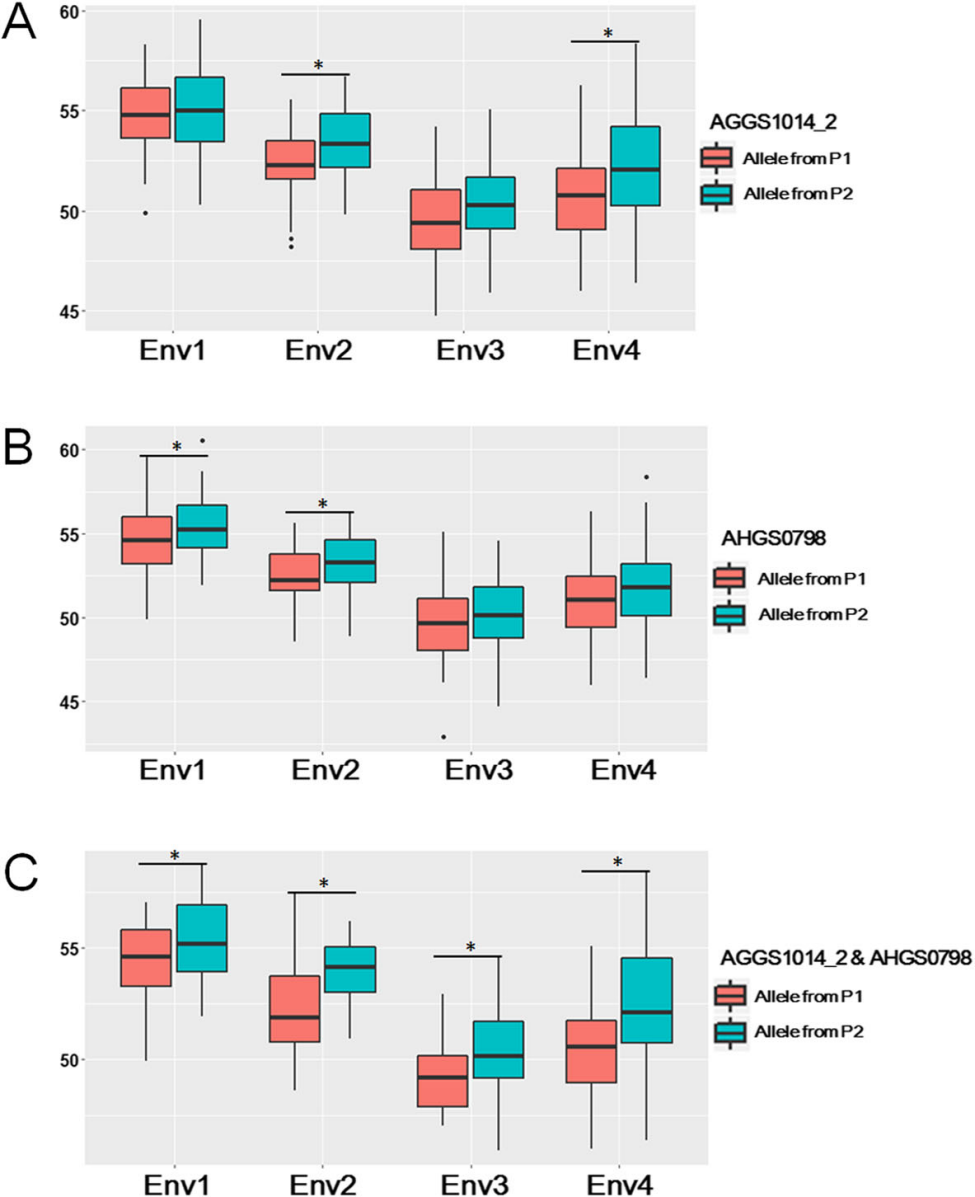
Confirmation of two trait-associated markers in a RIL population. Env1, 2, 3 and 4 represented field trials in Wuhan (2015), Wuhan (2016), Xiangyang (2017) and Wuhan (2017). P1 female parent, P2 male parent

To estimate potential values of associated loci in peanut breeding, a RIL population derived from two additional accessions (Zhonghua 10 and ICG12625) was employed as a test population. Oil content of the RIL population across four environments ranged from 47.45 to 60.88% in Env1, 45.30 to 58.96% in Env2, 42.89 to 55.07% in Env3, and 45.98 to 58.37% in Env4, respectively. The oil content of the female parent was 51.88 ± 1.41%, whereas that of the male parent was 53.32 ± 1.47%. Three makers (AGGS1014_2, AHGS0798, and AHGS1431) showed association with oil content in the RIL population. A significant difference in oil content between homozygous alleles from P1 and P2 at AHGS1431 locus was observed in Env1 (Additional file 1: Table S4). Compared with the homozygous allele from P1 at AGGS1014_2 locus, the homozygous allele from P2 had significantly higher oil content in two environments i.e., Env2 and Env4 (Fig. 4a and Additional file 1: Table S4). For marker AHGS0798, oil content of the homozygous allele from P2 was significantly higher than that of the homozygous allele from P1 in two environments (Fig. 4a and Additional file 1: Table S4). Combined allele effect of AGGS1014_2 and AHGS0798 showed that oil content of homozygous alleles from P2 was significantly higher than that of the homozygous allele from P1 across environments (Fig. 4c).

Among the 292 peanut accessions, the alleles at AGGS1014_2 (X) locus and AHGS798 (Y) locus formed six combined genotypes, namely X-205 bp/Y-170 bp, X-205 bp/Y-172 bp, X-205 bp/Y-174 bp, X-215 bp/Y-170 bp, X-215 bp/Y-172 bp, X-215 bp/Y-174 bp (Additional file 2: Fig. S5). The oil content is highest in X-215 bp/Y-174 bp (51.49 ± 1.30%), median in X-215 bp/Y-172 bp (51.04 ± 1.11%), X-215 bp/Y-170 bp (50.88 ± 1.17%), X-205 bp/Y-174 bp (50.66 ± 1.38%), and X-205 bp/Y-170 bp (49.72 ± 2.10%), and lowest in X-205 bp/Y-172 bp (49.62 ± 1.19%). The genotypic frequency of X-215 bp/Y-174 bp was 4.00% in peanut varieties released before 1980, and it increased to 22.13% in peanut varieties released after 2000. Similarly, the frequency of X-205 bp/Y-172 bp has an increase from 12.00% in peanut varieties released before 1980 to 32.79% in peanut varieties released after 2000. The frequency of X-205 bp/Y-172 bp and X-215 bp/Y-170 bp were lower in peanut varieties released after 2000 than the varieties released before 1980.

## Discussion

For performing high resolution mapping using GWAS studies, multilocation phenotyping data for target traits on diverse panel together with genotyping data is necessary for discovery of significantly associated markers. Keeping this in mind, this research effort used the peanut panel which consists of 222 cultivars, 55 breeding lines, and 15 landraces representing 17 provinces of China. The screening of genome-wide SSR marker in the peanut panel produced high number (3663) of alleles including 629 unique alleles showing high molecular diversity. For example, this panel of 292 Chinese cultivated accessions showed on par average allele number (2.99 to 8.10 per locus), gene diversity (0.51) and PIC (0.45) as compared to average allele number (6.28 per locus), gene diversity (0.11 to 0.59) and PIC (0.21 to 0.53) in other Chinese germplasm collections or US peanut mini-core collection [26–28]. On the other hand, the higher values for average allele number (22.21), gene diversity (0.74) and PIC (0.72) was observed in the peanut ‘reference set’ of ICRISAT [23] which may due to the diverse genotypes included from 48 countries representing global diversity including wild accessions. From all these comparisons, the Chinese cultivated accessions in the present study represented high molecular diversity comparable to other such collections consisting of cultivated genotypes, indicating that this population is suitable for association mapping. Several studies in other crops also reported that the genetic diversity of cultivated species was always lower than the corresponding wild species [29–32]. It is essential to deploy diverse and wild genetic resources into Chinese cultivars to broaden their genetic base of founder parents for enhancing the genetic diversity and achieving higher genetic gains. The information available through genotyping and multilocation phenotyping will further facilitate identification of potential founder parents for the ongoing breeding program.

Population structure is an important component in association mapping analysis and it helps in reducing the detection of false positives among associated markers. The STRUCTURE analysis identified two subpopulations for the 292 accessions (Fig. 1b) which had also been indicated by the dendrogram tree and PCA analysis (Fig. 1c and d). The peanut germplasm collections in previous studies could be divided into 2 to 4 subpopulations, which were always associated with the types of botanical varieties [23, 27, 33–35]. In the present study, the landraces in the peanut panel could be clearly divided into subsp. *hypogaea* (G1) and subsp. *fastigiata* (G2), respectively (Fig. 1c). However, most peanut cultivars and breeding lines in this population harbored mixed morphological features from the reciprocal cross between different botanical varieties. Thus, it is hard to distinguish the botanical difference between two subgroups clearly. Most accessions in the G1 group were from the provinces distributed on Northern China and the Yangtze River region. More than half of accessions in the G2 group were from the provinces distributed in Southern China (Fig. 2a and Additional file 1: Table S5). Comparing to Southern China, the varieties from Northern China were more closely related to the varieties from the Yangtze River region (Fig. 2b). It is indicated that the geographic origins of accessions had a significant effect on the population structure. A similar phenomenon was observed in many other crops [29, 36–38]. Different climate condition and their corresponding cropping system among Northern China, Southern China, and the Yangtze River region, might be responsible for genetic differentiation of the peanut population in China, enabling peanut varieties to adapt to various ecological environments.

**Fig. 5:**
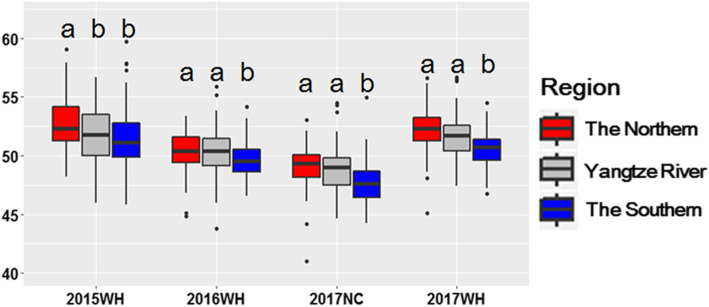
Comparison of oil content (%) among peanut accessions from different geographic regions. The boxes with different letters were significantly different according to Tukey’s Multiple Comparison Test (*P* < 0.05)

Oil content is an important trait in peanut breeding with polygenic inheritance. In the present study, the association analysis was performed to evaluate the phenotypic effect of multiple alleles in the diverse genetic background across multiple environments. A total of 42 alleles for twelve associated loci, explaining 4.54–9.94% phenotypic variance, were identified for oil content (Table 3). Interestingly, the favorable alleles with relatively higher effects were comparatively abundant in the varieties from Northern China and the Yangtze River region, than the varieties from Southern China (Figs. 3b and 4c). Correspondingly, oil content of varieties showed a geographic difference clearly. The accessions from Northern China and the Yangtze River region had significantly higher oil content than the accessions from Southern China (Fig. 5). It seemed that oil content and its underlying loci may undergo selection during geographical differentiation in China. However, more experimental evidence, such as a multiple-ecological investigation of phenotype, was needed to verify the hypothesis.

The oil content showed an additive inheritance in crops [15, 39–41], which could facilitate pyramiding associated loci straightforward in the breeding program. In this study, associated markers were widely located on chromosome A03, A04, A08, A09, A10, B01, B06, B07, and B08 (Additional file 1: Table S1) based on the information from previous linkage maps and the physical location on genome [25, 42–44]. Compared with the previous results, chromosome A03, A04, A08, B06, B07, and B08 were also found to harbor QTLs for oil content [13, 14, 16]. For instance, the associated marker AHGS0798 on chromosome B06 (124.9 Mb), is close to *q*OCB06.1 (121.9 Mb–124 Mb) detected in the RIL population derived from Xuhua 13 and Zhonghua 6 [16]. Another two markers (TC1A02 and AHGS0393), which were highly linked to QTLs for oil content in the early studies [13, 14], were located at 127.5 Mb and 139.3 Mb on chromosome B06. These results suggested that AHGS0798 with PVE of 7.28% would be a reliable marker associated with oil content. In addition, three associated markers (AGGS1014_2, pPGPseq8D9, and TC11B4_2) in the present study could not collocate with the previous QTLs, suggesting that they are newly identified loci controlling oil content. Among them, the locus (AGGS1014_2) was repeatedly detected in three environments, with the maximum –LogP value of 2.50E-06 and PVE of 9.94% (Table 3). To evaluate the potential value of these loci in peanut breeding, two associated markers (AHGS0798 and AGGS1014_2) were verified in the RIL population derived from Zhonghua 10 and ICG12625. The favorable allele of single locus (AGGS1014_2 or AHGS0798) could increase oil content by ~ 0.34% - ~ 1.50% or ~ 0.61% - ~ 0.88% in four environmental trials. Combining favorable alleles for two loci, oil content could increase to ~ 1.11% - ~ 2.06% (Fig. 4 and Additional file 1: Table S4). It is indicated that using associated markers to accumulate favorable alleles would be an effective way to increase oil content in peanut breeding. In the present peanut panel, AGGS1014_2-215 bp/AHGS0798-174 bp is one of six combined genotypes between two associated markers, which expressed the highest phenotypic effect. In the varieties released before 1980, one accession (4%) possessed this genotype whereas the frequency increased to 22.13% (27 accessions) in the varieties released after 2000 (Additional file 2: Fig. S5). It is suggested that the selection of favorable alleles of AGGS1014_2 and AHGS0798 has been underway in China during the breeding program.

## Conclusions

This study provided a resource to improve our understanding the genetic basis of oil content in peanut and to reveal a close relationship of the geographical region with population structure. Two associated markers (AGGS1014_2 and AHGS0798) in the present study were verified and can be deployed for use in GAB for enhancing oil content in peanut.

## Methods

### Plant materials and field planting

A total of 222 cultivars, 55 breeding lines, and 15 landraces from 17 different provinces in China were selected to constitute the peanut association mapping panel (Additional file 1: Table S5). A RIL population was developed from a cross between Zhonghua 10 and ICG12625 using single seed descent method and was later used for performing validation of associated markers. The F_8_–F_10_ generations of RIL population were used for analysis of oil content, the F_11_ generation was utilized for generation of genotypic data.

The peanut panel was planted in the experimental field of the Oil Crops Research Institute of the Chinese Academy of Agricultural Sciences in Wuhan from 2015 to 2017 and also in the experimental field of the Institute of Nanchong Agricultural Science and Technology in Nanchong in 2017. The RIL population was planted in the experimental field of the Oil Crops Research Institute of the Chinese Academy of Agricultural Sciences in Wuhan from 2015 to 2017 and in the experimental field of the Xiangyang Academy of Agricultural Science in Xiangyang in 2017. Field trials were conducted in a randomized complete block design with three replications. Each replication contained 12 plants at a spacing of 20 cm × 30 cm. Field management followed standard agricultural practice.

### DNA isolation and genotyping

The genomic DNA of 292 peanut accessions was extracted from fresh leaves following the modified cetyltrimethylammonium bromide method. The quality and quantity of DNA were checked using 1% agarose gel and NanoDrop (Thermo Fisher Scientific, USA), respectively.

Sixteen accessions with abundance phenotypic variation in peanut panel were used to screen polymorphic markers in 4485 previously reported markers [45–53]. A total of 583 high-quality polymorphic markers were obtained and labeled with fluorescence dyes to perform PCR amplification. The PCR production mixed with GeneScan 500 LIZ standard (Applied Biosystems, USA) was loaded to perform capillary electrophoresis using 3730 DNA Analyzer (Applied Biosystems, USA). The output of electrophoretic data was visualized and transferred to allele size using GeneMarker V2.1 software (https://softgenetics.com/GeneMarker.php). The SSR allele was numerically coded according to the fragment size.

### Genotypic data analysis

The allele number, major allele frequency, genetic diversity and polymorphism information content (PIC) were calculated using PowerMarker V3.25 software [54]. The number of subgroups of this peanut panel was estimated using STRUCTURE software V2.1 based on the model-based Bayesian clustering method [55]. To determine an optimum number of subgroups (*K*), five independent runs were performed to estimate each *K* values from 1 to 10 for each accession. For each run, a burn-in length of 50,000 followed by 10,000 iterations were conducted with the admixture and related frequency models. The optimal *K* value was determined by the posterior probability [LnP(D)] and Δ*K* [56].

Phylogenetic analysis was performed to construct a UPGMA tree based on Nei’s (1972) genetic distance. Nei’s (1972) genetic distance was calculated using PowerMarker [54] and the tree was formed using MEGA 4.0 (http://www.megasoftware.net). Principal component analysis (PCA) was complement using R package “FactoMineR” (https://cran.r-project.org/web/packages/FactoMineR/index.html) and three-dimensional scatter plot of PCA was completed using R package “scatterplot3d” (https://cran.r-project.org/web/packages/scatterplot3d/).

SSR markers mapped on a dense genetic map were selected to estimate LD. The pairs of markers located on the same linkage group were treated as linked markers, otherwise as unlinked markers. The *r*^*2*^ and *p* value was calculated with TASSEL 3.0 [57]. LD decay in the peanut panel with *r*^*2*^ values were plotted against the genetic distance (cM) between markers.

### Evaluation of oil content and phenotypic data analysis

The percentages of Oil and H_2_O in seeds were measured using nuclear magnetic resonance (PQ001, Niumag, China). Matured seeds (~ 10 g) with less than 10% moisture content were analyzed for each of the three sub-samples per entry. Oil content (%) was calculated based on dry-weight using the formula {[oil/(100 − H_2_O)] × 100} [13].

The field trials in Wuhan in 2015, 2016 and 2017 were treated as Environment I, II and III, respectively. The field trial in Nanchong in 2017 was treated as Environment IV. The phenotypic data statistical analyses were performed using the IBM SPSS Statistics software (V.22, IBM, USA). The family-based broad-sense heritability for oil content was calculated as ${H}^2={\sigma}_g^2/\left({\sigma}_g^2+{\sigma}_{g\times e}^2/r+{\sigma}_{\varepsilon}^2/ rn\right)$, where ${\sigma}_g^2$ is the genotypic variance, ${\sigma}_{g\times e}^2$ is the genotype × environment interaction variance, ${\sigma}_{\varepsilon}^2$ is the residual variance, r represents the number of environments and n represents the number of replications in each environment.

### Marker-trait association analysis

Associations between SSR markers and the trait of oil content were performed using TASSEL software based on a Q + K mixed linear model [57]. The population structure (Q) was obtained from model-based program STRUCTURE V2.1 [55]. The pairwise kinship matrix (K) was calculated using SPAGeDi software [58]. To estimate allelic effect, the phenotypic effect of last allele for an associated marker is set to zero and the other allele estimates are relatives to that. The mean of four-trial allelic effect at 12 association locus were used to construct a heatmap to view geographic distribution of allelic effect for associated markers. The software MeV was used to visualize the heatmap [59].

## Supplementary information

**Additional file 1: Table S1.** Statistic summary of 578 polymorphic markers. **Table S2.** Summary of allele frequency in 292 accessions. **Table S3.** Allelic effect of associated loci. **Table S4.** Comparison of oil content between two genotypes of three markers in ZI population. **Table S5.** The detailed information on 292 peanut accessions.

**Additional file 2: Figure S1.** Linkage disequilibrium (LD) decay in 292 peanut accessions. **Figure S2.** Description of phenotypic values for 292 peanut accessions. **Figure S3.** Comparison of oil content (%) among peanut accessions released at different stages. **Figure S4.** Association study for oil content. **Figure S5.** Frequency and phenotypic effect of combined genotypes between AGGS1014_2 and AHGS0798 in the peanut panel.

## Data Availability

The raw phenotype data and genotype data are available from the corresponding author on reasonable request.

## Abbreviations

Coefficients: *r*^*2*^
GAB: Genomics-assisted breeding
LD: Linkage disequilibrium
PCA: Principal component analysis
PIC: Polymorphic information content
PVE: Phenotypic variance explained
RIL: Recombinant inbred line
SSR: Simple sequence repeats
