# Supplementary material for: Dissection of the genetic basis of oil content in Chinese peanut cultivars through association mapping
Source: BMC Genet. 2020 Jun 8;21:60. doi: 10.1186/s12863-020-00863-1 (PMC7282078; doi:10.1186/s12863-020-00863-1)
Supplement: Supplementary file 2 — Additional file 2: Figure S1. Linkage disequilibrium (LD) decay in 292 peanut accessions. Figure S2. Description of phenotypic values for 292 peanut accessions. Figure S3. Comparison of oil content (%) among peanut accessions released at different stages. Figure S4. Association study for oil content. Figure S5. Frequency and phenotypic effect of combined genotypes between AGGS1014_2 and AHGS0798 in the peanut panel. [file 12863_2020_863_MOESM2_ESM.docx]

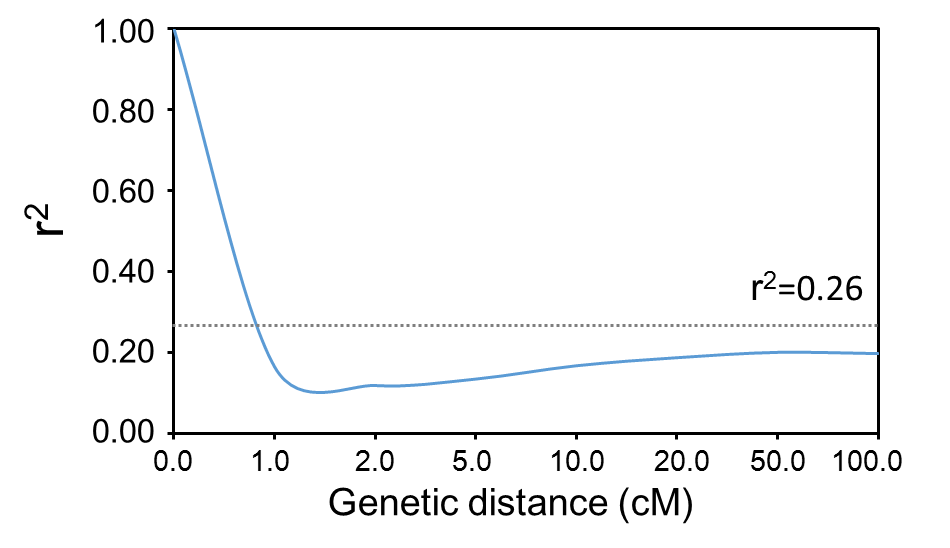


**Figure S1.** Linkage disequilibrium (LD) decay in 292 peanut accessions.


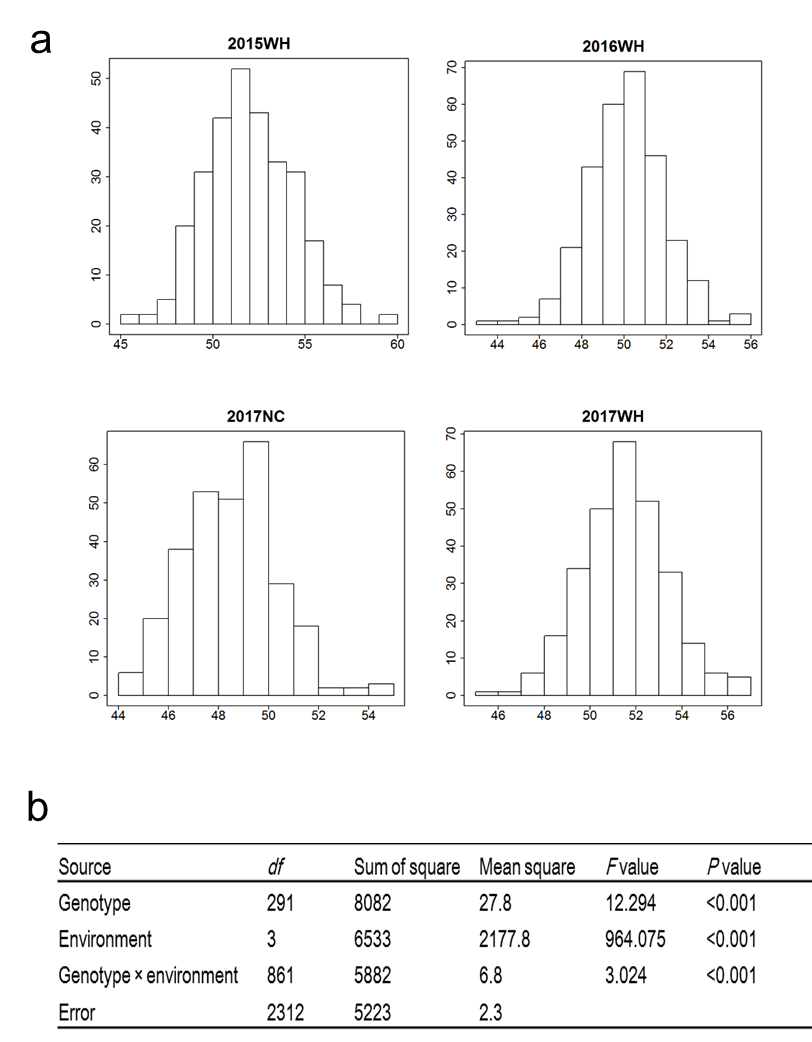


**Figure S2.** Description of phenotypic values for 292 peanut accessions. **a** Distribution of oil content across four environments. The x-axis represented oil content (%), the y-axis represented the number of individuals. **b** Analysis of variance for oil content across multiple environments.


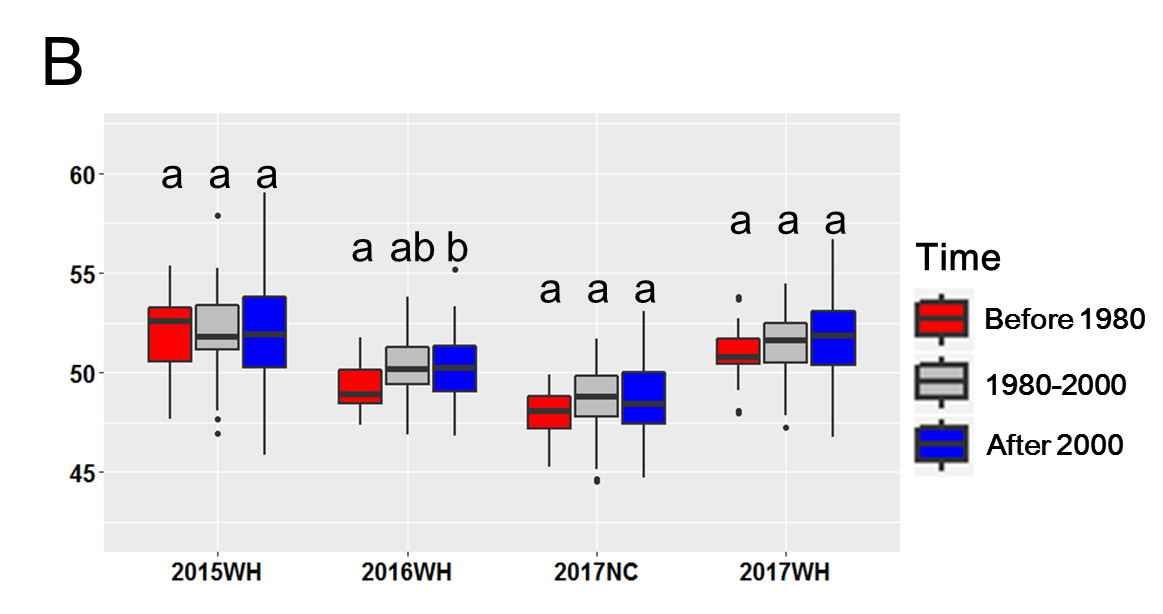


**Figure S3.** Comparison of oil content (%) among peanut accessions released at different stages. The boxes with different letters were significantly different according to Tukey's Multiple Comparison Test (*P* < 0.05)


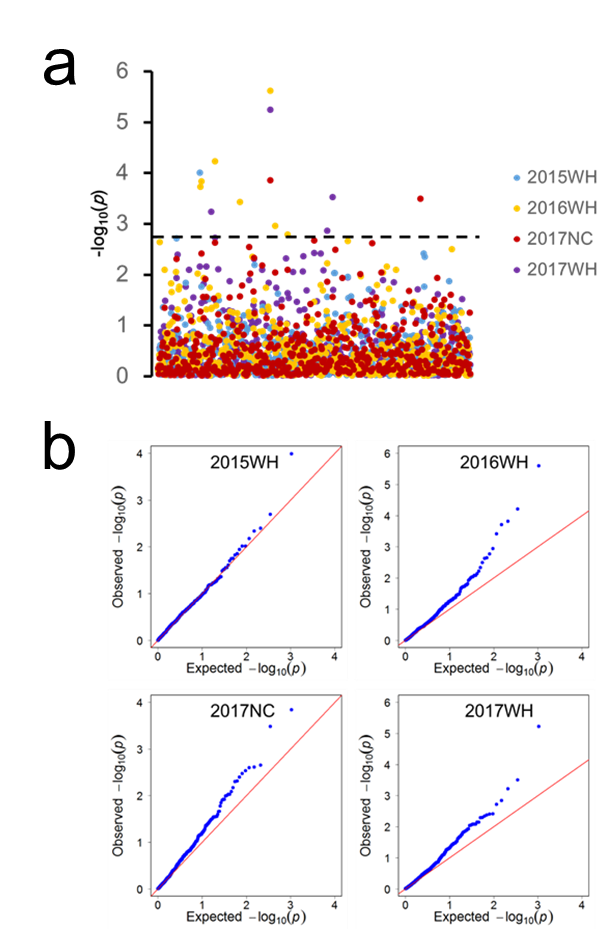


**Figure S4.** Association study for oil content. **a** Manhattan plot. The dashed line indicated the threshold of selection signals. **b** Quantile-Quantile plots. 2015WH, 2016WH, and 2017WH indicated 2015, 2016, and 2017 trial in Wuhan, respective. 2017NC indicated 2017 trial in Nanchong.


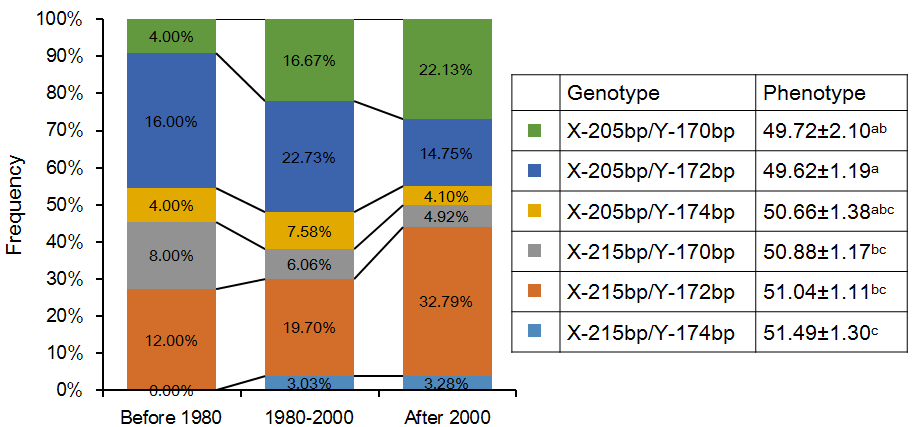


**Figure S5.** Frequency and phenotypic effect of combined genotypes between AGGS1014_2 and AHGS0798 in the peanut panel. X locus represented associated marker AGGS1014_2, Y locus denoted associated marker AHGS0798. Before 1980, 1980-2000, and After 2000 indicated peanut varieties released before 1980, at 1980-2000 and after 2000, respectively. In phenotype column, values that were not followed by the same letter were significantly different according to Tukey's Multiple Comparison Test at 0.05 level.
